# Milk Calcium and Phosphorus in Ugandan Women with HIV on Tenofovir-Based Antiretroviral Therapy

**DOI:** 10.1177/08903344221146472

**Published:** 2023-01-30

**Authors:** Florence Nabwire, Matthew M. Hamill, Mary Glenn Fowler, Adeodata Kekitiinwa, Ann Prentice

**Affiliations:** 1 MRC Nutrition and Bone Health Research Group, Cambridge, UK; 2 MRC Epidemiology Unit, University of Cambridge, Cambridge, UK; 3 Johns Hopkins University School of Medicine, Baltimore, MD, USA; 4 Baylor College of Medicine Children’s Foundation – Uganda (Baylor-Uganda), Kampala, Uganda

**Keywords:** Africa, antiretroviral therapy (ART), HIV, lactation, mother-to-child transmission, milk composition, tenofovir, Uganda

## Abstract

**Background:**

Breastfed infants depend on human milk calcium and phosphorus for bone mineral accretion and growth. We reported greater mobilization of bone mineral and delayed skeletal recovery in lactating Ugandan women with HIV initiated on tenofovir-based antiretroviral therapy during pregnancy compared to HIV-uninfected counterparts in the Gumba Study. However, it is unknown if these disruptions in maternal bone metabolism affect milk mineral concentrations.

**Research Aim:**

To compare concentrations and patterns of change in milk calcium and phosphorus between lactating women with and without HIV.

**Methods:**

A longitudinal observational study was conducted to compare milk mineral concentrations between women with HIV receiving tenofovir-based ART and uninfected women in the Gumba Study. Milk collected at 2, 14, 26, and 52 weeks lactation was analyzed for calcium and phosphorus. Sodium and potassium were measured at 2 and 14 weeks to detect sub-clinical mastitis. Differences in milk composition between 84 women with HIV and 81 uninfected women were investigated.

**Results:**

Women with HIV had higher milk calcium than uninfected women at 14 weeks. The percent difference was +10.2% (*SE* = 3.0, *p* = .008) and there was a tendency to greater values at 2 and 26 weeks. Milk calcium decreased in both groups during lactation (*p* ≤ .001) but was more pronounced in women with HIV. The magnitude of change within individuals in the 1st year of lactation from 2 to 52 weeks was −28.3% (*SE* 3.9) versus −16.5% (*SE* 3.5), *p* for interaction = .05. Differences in milk phosphorus and calcium-to-phosphorus ratio were smaller and mostly not significant.

**Conclusions:**

Participants with HIV on tenofovir-based antiretroviral therapy had altered milk mineral composition. Studies are needed to investigate mechanisms and health implications for the woman and infant.

**Key Messages**

- The effects of metabolic disturbances experienced by lactating women with HIV receiving tenofovir-based antiretroviral therapy on their milk calcium and phosphorus contents are unknown.
- Ugandan women with HIV on tenofovir-based antiretroviral therapy had higher milk calcium in the first months of lactation and a greater overall reduction in the first year of lactation than women without HIV.
- We have provided evidence that HIV and tenofovir-based antiretroviral therapy are associated with alterations in milk mineral composition, although the mechanisms require investigation.
- This is the first report of human milk calcium concentrations affected by extrinsic factors, with potential influences on the health of the lactating woman and growth of the infant.

## Background

Calcium (Ca) and phosphorus (P) are the main bone-forming minerals and must be acquired from the diet. Therefore, human milk is the main source of Ca for predominantly breastfed infants in the first 6 months of life, and later during complementary feeding, in resource-limited settings where diets are low in Ca (Prentice et al., 1999). In healthy women, about 200 mg of Ca (up to 400 mg in some individuals) are transferred daily into milk at peak lactation (around 10–12 weeks postpartum) to meet requirements for infant bone mineral accretion (Kovacs, 2016; Olausson et al., 2012). Ca and P secreted in human milk are derived from the diet and from maternal bone mobilization, as evidenced by temporary decreases in maternal bone mineral density (BMD). In general, maternal bone mineral mobilization is highest during the first 3–6 months of lactation, and BMD is generally fully recovered by 3 months after breastfeeding cessation (Kovacs, 2016; Olausson et al., 2012).

The World Health Organization (WHO, 2015, 2016) recommends exclusive breastfeeding for the first 6 months of life and continued breastfeeding for at least 2 years. This advice includes women with Human Immunodeficiency Virus (HIV) and those living in resource-limited settings. The WHO also recommends HIV testing and immediate initiation of lifelong antiretroviral therapy (ART) for all pregnant and lactating women with HIV to prevent mother-to-child transmission of HIV. Initiation of ART in non-pregnant individuals is accompanied by a 2%−6% decrease in BMD during the 1st year, especially with regimens containing tenofovir disoproxil fumarate (TDF) (Hamill et al., 2017). We recently reported, for the first time, greater bone mobilization during lactation and only partial skeletal recovery at 3 months post-lactation in Ugandan women with HIV (WWH) initiated on ART during pregnancy, compared to women without HIV participating in the Maternal ART and Bone (MAB) Study, known as the Gumba [Bone] Study in Uganda (Nabwire et al., 2020).

Poor growth outcomes in breastfed infants born to WWH on ART in Sub-Saharan Africa have been reported (Aizire et al., 2020), and it is known that maternal ART is transferred in small quantities into human milk (Benaboud et al., 2011). It is possible that the HIV/ART-associated alterations in bone metabolism we reported during lactation among WWH may also affect milk Ca-P metabolism and milk mineral composition. Therefore, the aim of the current analysis was to compare concentrations and patterns of change in milk Ca and P between WWH and women without HIV in the Gumba Study.

## Methods

### Research Design

A longitudinal observational study was conducted. The rationale for this design was two-fold. First, we had shown that WWH in the Gumba Study had greater lactational bone mobilization than women without HIV, suggestive of alterations in Ca and P metabolism that could affect milk composition. Second, because human milk composition varies across the lactational period, mineral analysis of milk collected longitudinally in the same individuals would be required in order to observe differences in concentration and chart changes during lactation between the two groups of women. Ethics and protocol approvals were obtained from the Institutional Review Boards of the Joint Clinical Research Centre (JCRC) and of Mulago Hospital (approval dates: June 26 and August 25, 2014), and the Uganda National Council for Science and Technology (UNCST, study number: 1680). Full details of the study, consent processes, and ethical approvals have been published (Nabwire et al., 2020).

The primary outcome variable for the Gumba Study was change in lumbar spine BMD. The sample size of 100 participants per group was calculated to detect at least a 2% group difference in mean change in BMD between L2 and L14 (2 and 14 weeks of lactation respectively) at 80% power and 0.05 significance (Nabwire et al., 2020). Human milk Ca concentration was defined a priori as a secondary outcome variable. Based on milk Ca concentrations measured in Gambian women at 3 months postpartum (mean 5.85 [*SD* 0.6] mmol/L) (Jarjou et al., 2006) and an assumption of 20% participants not available for milk collection (i.e. *n* = 80 per group), the sample size was sufficient to detect a group difference in milk Ca concentration at L14 of 0.26 mmol/L at 80% power and 0.05 significance.

### Setting and Relevant Contexts

The Gumba Study was conducted at the antenatal, postnatal, and HIV clinics within the Mulago National Teaching and Referral Hospital (Mulago Hospital) complex in Kampala, the capital city of Uganda. The clinics mainly served urban low- and middle-income households and services were provided free of charge. National guidelines at the time the study was conducted included recommendations for opt-out routine HIV-testing for all pregnant women and immediate initiation of lifelong ART (one first-line regimen comprised of TDF, lamivudine [3TC] and efavirenz [EFV]) in those diagnosed with HIV (Uganda Ministry of Health, 2013, 2016).

Prevalence rates from surveillance surveys of pregnant women attending sentinel antenatal clinics at the time of the study ranged from 4.8% to 13.5% (Uganda AIDS Commission, 2016). At a national level, the burden of HIV was higher in women than men (7.5% vs. 4.3%), and higher in urban areas compared to rural areas (7.1% vs. 5.5%; Uganda Ministry of Health, 2017). At that time, 97% of HIV-infected pregnant women in Uganda received ART for prevention of mother-to-child transmission (Uganda AIDS Commission, 2016).

In line with prevailing national infant and young child feeding guidelines at the time of the study, all women were advised to breastfeed their babies exclusively in the first 6 months of life, regardless of HIV status, and to continue breastfeeding for at least 12 months postpartum for WWH, and 24 months for women without HIV (Uganda Ministry of Health, 2013, 2016). National rates for exclusive breastfeeding in the first 6 months and continued breastfeeding at 18–24 months in the general population were 66% and 50%, respectively, at the time of conducting this study (Uganda Bureau of Statistics & ICF International, 2017).

### Sample

The target women for the longitudinal milk study were participants in the Gumba Study after parturition and the start of breastfeeding. The recruits into the Gumba Study were 100 WWH initiating TDF-based ART (previously ART-naïve) during pregnancy and 100 pregnant women without HIV in Kampala, Uganda. The cohort was followed until 3 months post-lactation. Pregnant women had been enrolled in the Gumba Study at 24–32 weeks gestation. To meet inclusion criteria, they had to be less than 36 weeks gestation with a documented rapid HIV test from Mulago Hospital during the index pregnancy using the nationally approved sequential rapid HIV testing algorithm. They had to be 18–39.9 years old with no known medical complications, planning to breastfeed for at least 6 months, and not planning to move away from Kampala in the following year. Exclusions at enrollment included a diagnosis of HIV prior to the index pregnancy, non-singleton pregnancy, classified as being at high-risk (hypertension, preeclampsia/eclampsia), or diagnosis of bone disease and conditions associated with abnormal bone metabolism and renal function (diabetes mellitus, gestational diabetes, tuberculosis, Hepatitis C, proteinuria, and renal disease). Later exclusions included preterm delivery (< 37 weeks gestation), stillbirth, neonatal death, breastfeeding cessation before 14 weeks postpartum, or subsequent pregnancy. All women included in the WWH group had been initiated on first line TDF-based ART regimen during the index pregnancy; those in the reference group (women without HIV) had a reported negative HIV test result.

**Figure 1.**
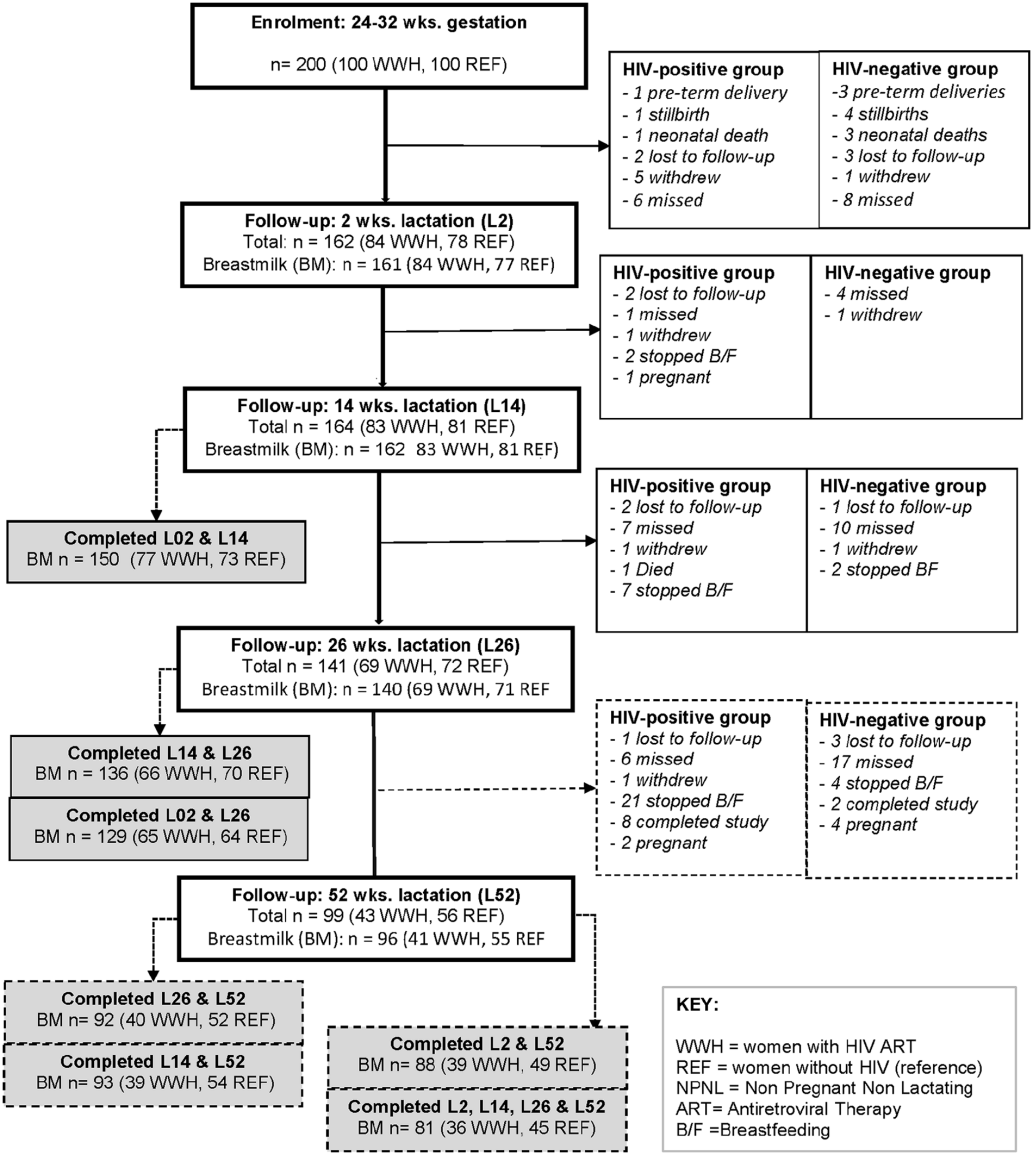
Flow of Participants in the Study and Numbers of Lactating Women Who Collected Milk Samples at Each Timepoint. *Note.* WWH = women with HIV initiated on lifelong triple tenofovir (TDF)-based ART during pregnancy (previously ART naïve); REF = women without HIV (reference group, ART naïve); L2, L14, L26, L52 = 2, 14, 26, and 52 weeks, respectively.

Figure 1 shows the overall flow of participants in the Gumba Study who provided milk samples during lactation. This details the reasons participants dropped out at various stages of the study, which were similar in both groups. Postpartum, 176 women (90 WWH and 86 women without HIV) participated in the Gumba Study at any timepoint. Milk samples were provided by 165 women on at least two occasions (84 WWH and 81 women without HIV). In all, 81 participants (36 WWH and 45 women without HIV) gave milk samples at all four timepoints (Supplement Table 1).

### Measurement

Participant characteristics were obtained using questionnaires and standard anthropometry. Detailed descriptions for anthropometry and other study measurements have been published (Nabwire et al., 2020). In brief, adherence to ART was based on the percentage of pills consumed divided by number of pills dispensed, and the WHO definition was used to define exclusive breastfeeding (EBF; yes vs. no). Other collected data about characteristics were prior use of the contraceptive depot medroxyprogesterone acetate (DMPA) before the index pregnancy (yes vs. no), current use of DMPA at any timepoint during the lactation period (yes vs. no) and resumed menses at any timepoint during the lactation period (yes vs. no).

The breastfeeding participants were invited to hand express 5 ml of milk from their left breast into separate plain blood tubes at L2, L14, L26, and L52. Prior to the collection, the participants were taught how to hand-express milk and were supported to do so when it was necessary. Milk samples were collected without special attention to time of day or stage of feed, because this has been shown to be unnecessary for studies of human milk Ca and P concentrations (Olausson et al., 2012). Milk samples were immediately placed in a cool box before storage at −80 °C within 2 hr, and later air freighted at −40 °C and then stored at −80 °C for laboratory analysis in Cambridge, United Kingdom.

Milk Ca and P contents were analyzed according to the semi-automated micro-method developed by Laskey et al. (Laskey et al., 1991). In summary, whole milk samples were warmed to 40 °C, and then aliquots of 20 µl prepared into acid-washed tubes. The tubes were then frozen, freeze-dried, and ashed in a muffle furnace at 400–500 °C. Next, the samples were cooled and digested with 0.5 ml of 0.2 mol/L hydrochloric acid. Ca and P in digested/prepared milk samples were then analyzed in duplicate on a Konelab analyser (Thermo Fischer Scientific, Vantaa, Finland) using Thermo Scientific kits for plasma calcium (Arsenazo III method) and phosphate (Ammonium molybdate method), respectively. Low, medium, and high control samples and materials supplied by kit manufacturers (Nortrol, Abtrol, Roche, and an internal drift control [LHQC]) were used for quality assurance. Mean coefficients of variation (CVs) for milk Ca and P concentrations were 0.9% and 0.8%, respectively.

Separate aliquots of whole milk collected at L2 and L14 were used for measurement of sodium (Na) and potassium (K) concentrations using flame photometry (IL 945 flame photometer). A total of 24 samples were analyzed with and without centrifugation, and the results were not significantly different (correlation coefficients were 0.98 and 0.99 for Na and K with no indication of bias). Therefore, Na and K were analyzed in whole milk without centrifugation. The flame photometer was calibrated using a standard urine control containing 73.5–81.8 mmol/L of Na and 27.8–34.4 mmol/L of K. The mean CVs for milk Na and K were 4.0% and 3.6%, respectively. Subclinical mastitis was defined as a milk Na-to-K ratio (Na:K) above 1.0 mmol/mmol (Kasonka et al., 2006).

### Data Collection

Milk samples were collected between March 2015 and February 2017 as per the approved study protocol for the Gumba Study, and laboratory analysis for milk composition was conducted in 2018. Follow-up visits were scheduled at the health facility at 36 weeks gestation, at 2 (L2), 14 (L14), 26 (L26), and 52 (L52) weeks of lactation, and at 14 weeks post-lactation. Participants were contacted in advance to remind them to attend study appointments. Compensation for travel costs and a snack were provided in line with prevailing national guidelines (Uganda National Council for Science and Technology, 2014).

Written informed consent was obtained from all participants in their own language before enrollment in the study and additional consent was obtained for storage and analysis of biological samples, including milk. To maintain confidentiality, all participants were assigned a study number which was used on all study forms and samples. Hardcopy and electronic study documents were stored under lock and key and password-protected files, respectively. Handling and processing of all study data complied with institutional processes, and U.K. data protection and Uganda national guidelines (UNCST, 2014).

### Data Analysis

For the purposes of the longitudinal study presented in this paper, data from participants who provided a milk sample on only one occasion were excluded from the analysis. Descriptive statistics for continuous variables are presented as either mean with standard deviation (*SD*) for normally distributed variables or median with 25^th^ percentile and 75^th^ percentile quartiles for skewed distributions. Descriptive statistics for discrete variables are presented as either proportions (%) with *SD*, or medians with 25^th^ percentile and 75^th^ percentile quartiles. Cross-sectional group differences at each timepoint were investigated using Analysis of Variance (ANOVA) for continuous variables and chi-square tests for proportions. Age, parity, primiparity, EBF, and prior and current use of hormonal contraception were investigated at each timepoint as potential confounders of differences in milk mineral concentrations between the groups. Data were analyzed using DataDesk 6.3.1 software (Data Description Inc, Ithaca, NY, USA).

For modeling longitudinal changes in milk composition, data were transformed into natural logarithms (log_e_) and multiplied by 100 before data analysis. Conversion of variables to natural logarithms normalized positively skewed data and, once multiplied by 100, enabled the differences between groups and changes within group to be expressed as sympercents ([difference/mean] × 100; Cole & Altman, 2017). Within-individual changes in milk composition over time in each group and differences between groups were investigated on these log_e_ transformed data using repeated measures ANOVA in 4-timepoint hierarchical/nested models constructed for each variable with an individual identifier (nested by group), timepoint, group, and a group-by-timepoint (group*visit) interaction term. Outputs from the models are expressed as mean percentage differences with standard errors of the mean (*SE*; Cole & Altman, 2017). Scheffé post-hoc tests in these hierarchical models were used to provide estimates of size and significance of between-group differences at each timepoint and within-group changes between each timepoint for each variable, and to account for multiple testing. All within-group changes and percentage differences between groups presented in this paper are from the 4-timepoint hierarchical models.

The 4-timepoint hierarchical models were used to test the significance of between group differences in the overall patterns of change between L2 and L52 (presented as *p* for 4-timepoint group*visit interaction terms). To test for between-group differences in change from one timepoint to another, a series of 2-timepoint hierarchical models were constructed and the significance of any difference judged by *p* for the group*visit interaction term for each pair. Three-timepoint hierarchical models were also developed to compare patterns of change in milk composition in WWH and women without HIV, first during the first 6 months of lactation (L2-to-L14-to-L26) when most women were breastfeeding exclusively and maternal bone mobilization was greatest (Nabwire et al., 2020), and second from L14 to L52 (L14-to-L26-to-L52) because of observed differences between groups at L14. The associated *p* for group*visit interaction terms in the 3-timepoint models for Ca, P and Ca-to-P ratio (Ca:P) are presented. Finally, a sensitivity test was performed by repeating the 4-timepoint models on data restricted to the 81 participants who provided milk samples at all four sampling occasions (rectangular dataset). Two-sided *p* of ≤ .05 was considered significant for all tests.

## Results

### Participant Characteristics

The characteristics at each timepoint for participants providing ≥ two milk samples during lactation are presented in Table 1. Overall, maternal characteristics for this subset were very similar to those previously reported for the whole Gumba Study cohort (Nabwire et al., 2020).

**Table 1.** Characteristics and medical history by group and timepoint for participants who collected ≥2 milk samples during lactation (*N* = 165).

	Time Point for Collecting Milk Samples							
	L2		L14		L26		L52	
Characteristic	WWH *n(%)*=80(95)	REF *n(%)*=74(91)	WWH *n(%)*=81(96)	REF *n(%)*=80(99)	WWH *n(%)*=69(82)	REF *n(%)*=71(88)	WWH *n(%)*=40(49)	REF *n(%)=*55(68)
Weeks postpartum^1^	2.2 (0.5)	2.1 (0.4)	14.3 (0.5)	14.2 (0.8)	26.4 (0.8)	26.6 (0.8)	52.2 (0.8)	52.6 (0.9)
Weeks on ART^1^	17.7 (5.5)	—	29.3 (5.2)	—	41.8 (5.7)	—	68.3 (5.8)	—
% pills taken^1, A^	99.4 (1.9)	—	99.7 (1.6)	—	99.4 (3.3)	—	99.6 (1.5)	—
CD_4_ cell count^2^	400 (294, 516)	—	403 (283, 539)	—	488 (340, 676)	—	496 (372, 705)	—
Age (years)^2^	23.8 (21.5,27.6)	23.3 (20.9,27.2)	23.9 (21.7,27.3)	23.6 (21.1,27.4)	24.2 (22.3,27.9)	23.7 (21.4,27.4)	25.1 (22.9,28.7)	24.3 (21.9,28.0)
Parity^2^	2 (1, 3)	1 (1, 2)	2 (1, 3)	1 (1, 2)	2 (1, 3)	1 (1, 2)	2 (1, 3)	2 (1, 2)
Primiparous %^3^	36.3^a^	52.7	37.0^a^	55.0	36.2^a^	54.9	37.5	47.3
EBF %^3^	82.5^c^	54.1	86.4^c^	63.8	87.0^c^	46.5	0	0
Prior DMPA %^3^	38.8^b^	20.2	35.8^b^	18.8	36.2^a^	21.1	40.0	23.6
Current DMPA %^3^	0	0	29.6^b^	13.8	34.8	21.1	42.5	25.5
Resumed menses %^3^	0	0	39.5	35.0	55.1	50.7	100	100

*Note.* L2, L14, L26, L52 = 2, 14, 26, and 52 weeks lactation, respectively; WWH = women with HIV initiated on tenofovir-based ART during pregnancy (previously ART-naïve) who provided ≥ 2 milk samples during lactation (*n* = 84); REF = participants without HIV who provided ≥ 2 milk samples during lactation (*n* = 81); *n%* = number (percentage) of participants providing a milk sample at the respective timepoint; ART = antiretroviral therapy; CD_4_ cell count = cells/cm^3^; EBF = exclusive breastfeeding; DMPA = depot medroxyprogesterone acetate.^1^Means (*SD*), ^2^medians (25 percentiles, 75 percentiles), ^3^percentage (%) of participants reporting “yes.” ^A^Mean % adherence to ART based on the pill count method used in routine clinical care = 100*[number of pills taken/number of pills dispensed for the duration].

a,b,c Two-sided *p* for differences between the groups (WWH vs. REF) obtained from chi-square tests. ^a^*p* ≤ .05. ^b^*p* ≤ .01. ^c^*p* ≤ .001.

### Differences Between Groups in Milk Mineral Concentrations

**Figure 2.**
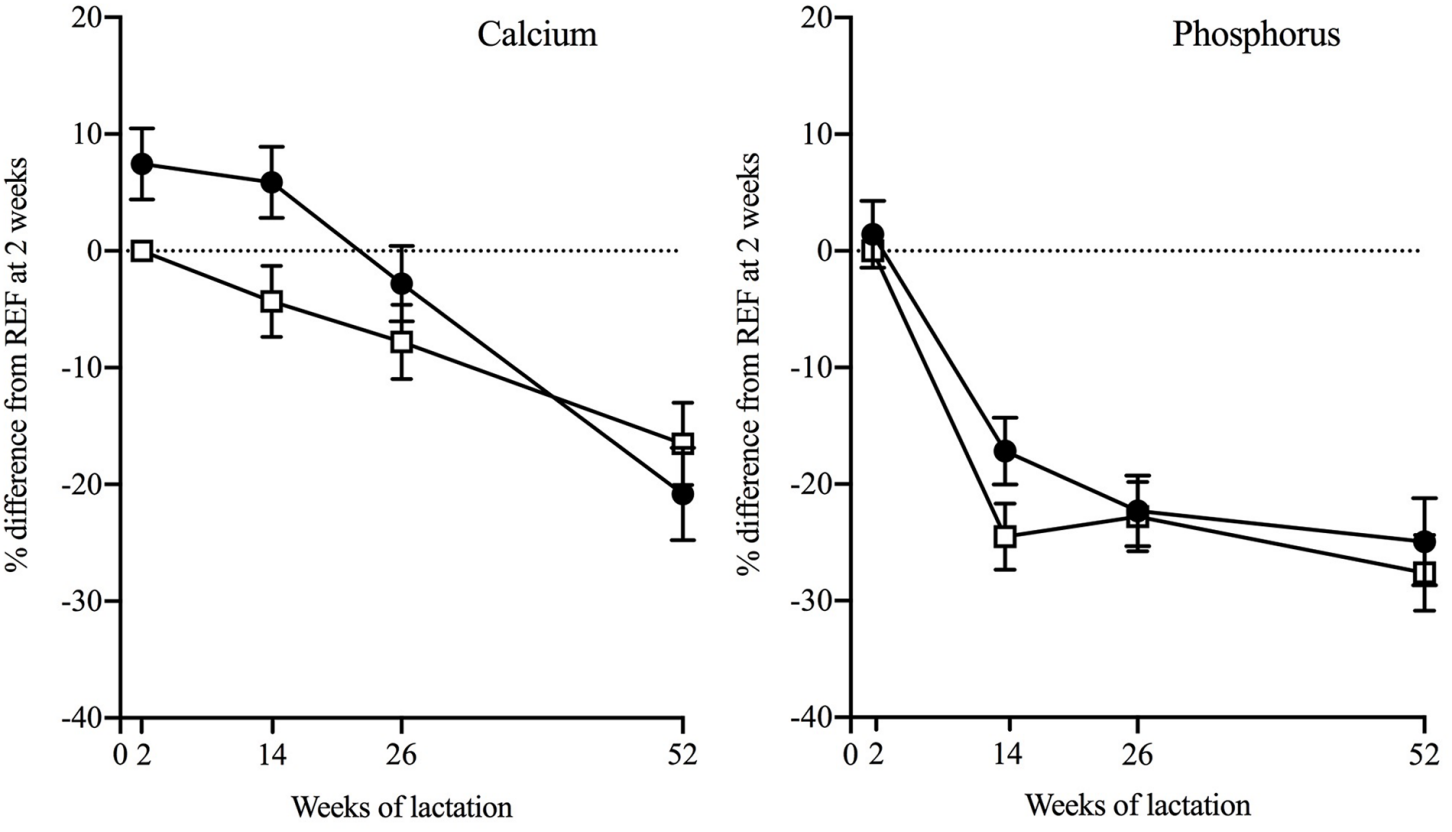
Differences Between Groups in Milk Calcium and Phosphorus Concentrations of Ugandan Women Across 52 weeks of Lactation. *Note.* Data are presented as mean percentage difference (standard error of the mean) from REF at 2 weeks from four-timepoint hierarchical models (*p*-forinteraction: calcium = 0.05; phosphorus = 0.34). Solid circles = women with HIV initiated to ART in pregnancy (WWH) who provided ≥ 2 milk samples during lactation (*n* = 84); open squares = women without HIV (REF) who provided ≥ 2 milk samples during lactation (*n* = 81).

Milk mineral concentrations by group at each timepoint are given in Table 2. Mean percentage differences between the groups at each timepoint from the 4-timepoint models are presented in Table 3. Mean milk Ca concentrations were higher in WWH than participants without HIV in the first months of lactation (Figure 2, Tables 2 and 3). There was also a tendency towards higher milk P in WWH at L14, so there was no significant difference in Ca:P ratio. Milk Ca, P and Ca:P ratio were similar in WWH and participants without HIV at L52. Of the potential confounders considered, EBF was a negative predictor of milk Ca, significantly so at L26 (EBF vs. non-EBF, mean difference was −8.7 %, [*SE* = 3.7; *p* = .02]). Because more WWH than participants without HIV were EBF except at L52 (Table 1), adjusting for EBF increased the magnitude and significance of the group differences in milk Ca at L14 and L26. The mean difference before adjustment at L14 between WWH versus participants without HIV was +10.0% [*SE* = 3.4; *p* = .005], at L26 it was +5.9% [*SE* = 3.1; *p* = .06]. After adjustment it was +11.0% [*SE* = 3.7; *p* = .003] at L14, and = +10.1% [*SE* = 3.5; *p* = .004] at L26.

**Table 2.** Milk Mineral Concentrations by Group and Timepoint for Participants (*N* = 165).

Concentration	Timepoints for Providing Milk Samples							
	L2		L14		L26		L52	
	WWH*n (%)* = 80(95)	REF*n(%)* = 74(91)	WWH*n(%)* = 81(96)	REF*n(%)* = 80(99)	WWH*n(%)* = 69(82)	REF*n(%)* = 71(88)	WWH*n(%)* = 40(49)	REF*n(%)* = 55(68)
Ca mmol/L^1^	4.83 (1.19)^a^	4.43 (0.97)	4.70 (0.96)^b^	4.29 (1.01)	4.33 (0.77)^b^	4.09 (0.80)	3.79 (1.12)	3.74 (0.83)
P mmol/L^1^	5.73 (1.14)	5.60 (1.05)	4.79 (0.99)^a^	4.45 (0.93)	4.53 (0.75)	4.45 (0.64)	4.53 (1.16)	4.35 (1.03)
Ca:P mmol/mmol^1^	0.85 (0.18)	0.80 (0.17)	1.01 (0.21)	0.98 (0.19)	0.98 (0.22)	0.94 (0.19)	0.87 (0.23)	0.87 (0.18)
Na,mmol/L^2^	7.40 (5.23, 10.3)	8.40 (6.33, 10.2)	4.35 (3.20, 5.70)	4.20 (3.30, 5.70)	—	—	—	—
K mmol/L^1^	16.8 (3.2)	16.5 (2.97)	13.4 (2.4)	12.9 (2.3)	—	—	—	—
Na:K mmol/mmol^2^	0.45 (0.30, 0.67)	0.46 (0.38, 0.63)	0.35 (0.26, 0.42)	0.33 (0.26, 0.45)	—	—	—	—
Na:K > 1.0^3^	10 (12.5%)	10 (13.5%)	6 (7.4%)	2 (2.5%)	—	—	—	—

*Note.* Values for Ca, P, Na and K are in mmol/L, ratios in mmol/mmol; to convert mmol to mg multiply Ca by 40, P by 31, Ca:P by 1.29, Na by 23, K by 39, Na:K by 0.59. Missing values at L2: WWH K = 1, Na/K = 1, REF Ca = 1, Ca:P = 1, Na =1, K = 1, Na:K = 1; at L14: WWH P = 1, Ca:P = 1, Na = 3, K = 3, Na:K = 3, REF Na = 4, K = 7, Na:K = 7; at L26: REF P = 1, Ca:P = 1; at L52: WWH Ca=1, P = 2, Ca:P = 3, REF Ca=2, P=2, Ca:P=2; milk Na and K were measured only at L2 and L14. L2, L14, L26, L52 = 2, 14, 26, 52 weeks postpartum, respectively; WWH = women with HIV initiated on tenofovir-based ART during pregnancy (previously ART-naïve) who provided ≥ two milk samples during lactation (*n* = 84); REF = participants without HIV who provided ≥ two milk samples during lactation (*n* = 81); *n*% = number (percentage) of participants providing a milk sample at the respective timepoint; Ca = calcium; P = phosphorus; Ca:P = calcium to phosphorus ratio; Na = sodium; K = potassium; Na:K = sodium to potassium ratio; Na:K > 1 = sodium to potassium ratio greater than 1 mmol/mmol. ^1^means (*SD*), ^2^medians (25 percentiles, 75 percentiles), ^3^number (%) of samples with Na:K > 1.

a,b Two-sided *p* for differences between the groups (WWH versus REF) obtained from ANOVA with adjustment for exclusive breastfeeding if significant: ^a^*p* ≤ .05, ^b^*p* ≤ .01.

**Table 3.** Percentage Differences in Milk Mineral Concentrations Between the Groups by Timepoint for Participants (*N* = 165).

Concentrations	WWH versus REFL2		WWH versus REFL14		WWH versus REFL26		WWH versus REFL52	
	%∆ (SE)	*p*	%∆ (SE)	*p*	%∆ (SE)	*p*	%∆ (SE)	*p*
Ca	+7.4 (3.1)	0.12	+10.2 (3.0)	0.008	+5.0 (3.3)	0.51	−4.3 (4.3)	0.80
P	+1.4 (2.9)	0.97	+7.3 (2.8)	0.08	+0.5 (3.1)	0.99	+2.6 (4.0)	0.93
Ca:P ratio	+6.4 (2.6)	0.12	+3.1 (2.6)	0.70	+3.3 (2.8)	0.71	−4.8 (3.7)	0.65
Na	−13.3 (8.0)	0.10	+8.9 (7.9)	0.27	—	—	—	—
K	+1.7 (2.8)	0.54	+4.2 (2.8)	0.14	—	—	—	—
Na:K ratio	−13.2 (8.6)	0.13	+5.4 (8.4)	0.52	—	—	—	—

*Note:* The + or − signs are used to indicate the direction of between-group changes (WWH higher or lower than REF, respectively). Results were obtained from Scheffé post-hoc tests in 4-timepoint hierarchical repeated-measures ANOVA models that included individual (nested by group), group, visit, and group*visit interaction. WWH = women with HIV initiated on tenofovir-based ART during pregnancy (previously ART-naïve) who provided ≥ two milk samples during lactation (*n* = 84); REF = participants without HIV who provided ≥ two milk samples during lactation (*n* = 81); L2, L14, L26, L52 = 2, 14, 26, 52 weeks postpartum, respectively; Ca = calcium; P = phosphorus; Ca:P = calcium to phosphorus ratio; Na = sodium; K = potassium; Na:K = sodium to potassium ratio; milk Na and K were measured only at L2 and L14. %∆ (*SE*) = mean percentage difference (*SE*) between the groups; *p* = significance of the difference (two-sided).

Milk Na, K and Na:K ratio measured at L2 and L14 were not significantly different between the groups at either timepoint (Tables 2 and 3). Prevalence of subclinical mastitis (milk Na:K ratio above 1.0) was 13.0% and 5.0% at L2 and L14 respectively and was not significantly different between the groups (Table 2). EBF was a significant negative predictor of milk Na and Na:K ratio at L14 (*p* = .03 and *p* = .05, respectively) but adjustment for EBF did not materially affect the differences between WWH and participants without HIV.

### Patterns of Change in Milk Mineral Concentrations in the 1st Year of Lactation

The patterns of change in milk Ca and P in the 1st year of lactation are illustrated in Figure 2. Mean within-individual percentage changes in WWH and participants without HIV across the timepoints from the 4-timepoint models are given in Table 4. The corresponding within-group changes between the intervening timepoints are given in Tables 5 and 6.

**Table 4.** Overall Magnitude of Changes in Milk Mineral Concentrations Within-Individuals During the First Year of Lactation by Participant Group (*N* = 165).

Mineral	WWHL2 to L52%∆ (SE)	REFL2 to L52%∆ (SE)	4-timepoint group*visitL2 to L14 to L26 to L52*p*-for-interaction^1^	2-timepoint group*visitL2 to L52*p-*for-interaction^2^
Ca	−28.3 (3.9)^a^	−16.5 (3.5)^a^	0.05	0.04
P	−26.4 (3.7)^a^	−27.6 (3.3)^a^	0.34	0.65
Ca:P ratio	+0.1 (3.4)	+11.3 (3.1)^b^	0.11	0.10

*Note.* The + or − signs are used to show the direction of the mean within-individual change in each group (increase or decrease, respectively). All data were transformed into natural logarithms and multiplied by 100 before data analysis. WWH = women with HIV initiated on tenofovir-based ART during pregnancy (previously ART-naïve) who provided ≥ two milk samples during lactation (*n* = 84); REF = participants without HIV who provided ≥ two milk samples during lactation (*n* = 81); L2, L14, L26, L52 = 2, 14, 26, 52 weeks lactation, respectively; Ca = calcium; P = phosphorus; Ca:P = calcium to phosphorus ratio; %∆ (*SE*) = mean percentage within-individual change (standard error of the mean) obtained from Scheffé post hoc tests from 4-timepoint hierarchical repeated-measures ANOVA models, that included individual identifier (nested by group), group, visit, and group*visit interaction. ^1^Two-sided *p* for group*visit interaction term in 4-timepoint hierarchical repeated measures ANOVA models, L2-L14-L26-L52. ^2^Two-sided *p* for group*visit interaction term in 2-timepoint hierarchical repeated measures ANOVA models L2–L52.

a,b Significance (two-sided) of within-individual changes between L2 and L52 from the Scheffé tests in the 4-timepoint model

a *p* ≤ .001, ^b^*p* = .004.

**Table 5.** Within-Individual Changes in Milk Mineral Concentrations Between Each Visit by Participant Group (*N* = 165).

Mineral	L2 to L14		L14 to L26		L26 to L52	
	WWH%∆ (SE)	REF%∆ (SE)	WWH%∆ (SE)	REF%∆ (SE)	WWH%∆ (SE)	REF%∆ (SE)
Ca	−1.6 (3.0)	−4.3 (3.1)	−8.7 (3.1)^a^	−3.5 (3.1)	−18.0 (3.9)^c^	−8.7 (3.5)
P	−18.6 (2.8)^c^	−24.5 (2.9)^c^	−5.1 (3.0)^A^	+1.7 (2.9)	−2.7 (3.7)	−4.8 (3.3)
Ca:P ratio	+17.2 (2.6)^c^	+20.6 (2.6)^c^	−3.8 (2.7)	−4.1 (2.7)	−13.4 (3.5)^2^	−5.2 (3.0)
Na	−46.6 (7.9)^c,A^	−68.7 (8.1)^c^	—	—	—	—
K	−22.1 (2.8)^c^	−24.5 (2.9)^c^	—	—	—	—
Na:K ratio	−24.4 (8.4)^b^	−43.0 (8.7)^c^	—	—	—	—

*Note.* Data were obtained from Scheffé post-hoc tests in 4-timepoint hierarchical repeated-measures ANOVA models, that included individual identifier (nested by group), group, visit, and group*visit interaction. The + or − signs are used to indicate the direction of within-group changes (increase or decrease, respectively). All data were transformed into natural logarithms and multiplied by 100 before data analysis. L2, L14, L26, L52 = 2, 14, 26, 52 weeks lactation, respectively; WWH = women with HIV initiated on tenofovir-based ART during pregnancy (previously ART-naïve) who provided ≥ two milk samples during lactation (*n* = 84); REF = participants without HIV who provided ≥ two milk samples during lactation (*n* = 81); %∆ (SE) = mean percentage within-individual change between the pair of timepoints (standard error of the mean); Ca = calcium; P = phosphorus; Ca:P = calcium to phosphorus ratio; Na = sodium; K = potassium; Na:K = sodium to potassium ratio.

A Significance (two-sided) of group*visit interaction term in the 2-timepoint model for the pair of timepoints *p* ≤ .05.

a,b,c Significance (two-sided) of within-individual changes in the group obtained from the Scheffé post-hoc tests in the 4-timepoint model. ^a^*p* ≤ .05, ^b^*p* = .005, ^c^*p* ≤ .001.

**Table 6.** Within-Individual Changes in Milk Mineral Concentrations Over Three Consecutive Visits by Participant Group (*N* = 165).

Mineral Concentrations	L2 to L26		L14 to L52		L2 to L14 to L26	L14 to L26 to L52
	WWH%∆ (SE)	REF%∆ (SE)	WWH%∆ (SE)	REF%∆ (SE)	3-timepoint group*visit*p-*for-interaction	3-timepoint group*visit*p-*for-interaction
Ca mmol/L	−10.3 (3.2)^a^	−7.8 (3.2)	−26.7 (3.9)^c,A^	−12.2 (3.4)^b^	0.43	0.05
P mmol/L	−23.7 (3.0)^c^	−22.8 (3.0)^c^	−7.8 (3.7)	−3.1 (3.2)	0.16	0.24
Ca:P ratio	+13.4 (2.7)^c^	+16.5 (2.8)^c^	−17.2 (2.4)^c^	−9.3 (3.0)^a^	0.61	0.06

*Note:* Data were obtained from Scheffé post-hoc tests in 4-timepoint hierarchical repeated-measures ANOVA models, that included individual identifier (nested by group), group, visit, and group*visit interaction. The + or − signs are used to indicate the direction of within-group changes (increase or decrease, respectively). All data were transformed into natural logarithms and multiplied by 100 before data analysis. The 3-timepoint group*visit *p*-for-interaction are from 3-timepoint models for each set of consecutive 3 timepoints (L2 to L14 to L26, L14 to L26 to L52 respectively). L2, L14, L26, L52 = 2, 14, 26, 52 weeks postpartum, respectively; WWH = women with HIV initiated on tenofovir-based ART during pregnancy (TDF-3TC-EFV, previously ART-naïve) who provided ≥ two milk samples during lactation (*n* = 84); REF = participants without HIV who provided ≥ two milk samples during lactation (*n* = 81); %∆ (SE) = within-individual mean percentage change between adjoining three timepoints and standard error of the mean; Ca = calcium; P = phosphorus; Ca:P = calcium to phosphorus ratio.

A Significance of group*visit interaction term in the 2-timepoint model for the outer pair of timepoints *p* ≤ .05.

a,b,c Significance of within-individual changes in the group obtained from the Scheffé post-hoc tests ^a^*p* ≤ .05, ^b^*p* = .006, ^c^*p* ≤ .001.

Milk Ca and P decreased in the 1st year of lactation in both WWH and participants without HIV (Table 4), while Ca:P ratio increased in the first 6 months and decreased thereafter to a value similar to or higher than at L2 (Tables 4 and 5). There was a significant difference between WWH and participants without HIV in the pattern of change in milk Ca (4-timepoint model, *p*-for-interaction *p* = 0.05), predominantly due to the higher concentrations in the first 6 months and a steeper decrease between L14 and L52 (Figure 2, Table 6). This was not reflected in significant group differences in patterns of change over 52 weeks in milk P or Ca:P, except for change in P between L14 and L26 (Table 5).

WWH had a smaller decrease in milk Na than participants without HIV between L2 and L14 (Table 5), but changes in K and Na:K were not significantly different between the groups. Excluding the eight participants at L14 with sub-clinical mastitis (Na:K >1.0) made little difference to the patterns of change in Ca and P within or between the groups.

Restricting the analysis to participants with milk measurements at all four timepoints (rectangular dataset) resulted in larger between-group differences and patterns of change in mineral composition than in the full dataset, but, as expected with the smaller numbers, their statistical significance was generally reduced or lost (see Supplement Tables 2 and 3 in the online Supplemental Material).

## Discussion

The current study was designed to compare milk mineral concentrations from Ugandan participants with HIV initiated on ART in pregnancy (WWH) with those from HIV-unaffected counterparts (referent group). The result was that differences were found in both the mean values and patterns of change in Ca concentrations, and to some extent P. Overall, we observed milk Ca concentrations in this cohort that were low (e. g., *M* = 4.50 mmol/L [180 mg/L] at 3 months) compared to many populations worldwide but similar to some in other parts of Africa (Prentice et al., 1999). Milk Ca gradually decreased in both groups consistent with previous studies (Olausson et al., 2012), reducing to a mean of 3.76 mmol/L (150 mg/L) at L52. However, WWH, compared to the referent group, had higher milk Ca concentrations in the first months of lactation followed by greater decreases between L14 and L52. To our best knowledge, this is the first report of alterations in milk calcium-phosphorus metabolism in WWH initiated on TDF-based ART during pregnancy under the current universal ART and infant feeding guidelines for WWH in resource-limited settings (WHO, 2015, 2016).

We have previously reported greater decreases in BMD in the first 6 months of lactation, suggestive of greater bone mineral mobilization, in WWH recently initiated onto TDF-based ART compared to the referent group in this cohort of Ugandan participants (Nabwire et al., 2020). Our finding of higher milk Ca in WWH in the first months of lactation suggested there was an influx of Ca into milk, coinciding with the greater mobilization of Ca from maternal bone. Previously, researchers have shown that milk Ca concentration is fairly constant in the first 3 months of lactation (after the colostrum phase) and independent of milk volume, timing within the feed, dietary Ca intake, and Ca supplementation (Olausson et al., 2012). Although, subclinical mastitis is associated with the temporary opening of paracellular pathways causing leakage of plasma components into human milk (Prentice et al., 1985), the Na:K ratio was not significantly different between the groups, suggesting that sub-clinical mastitis was not the cause of the observed influx of Ca into milk. Thus, the higher milk Ca in WWH during the first months of lactation is remarkable, although well within the range seen in participants around the world (Prentice et al., 1999). Future research will be needed to identify factors that affect milk Ca concentration and its variation between participants, both within and between populations, and in those with and without HIV.

The reasons for the observed differences in milk Ca between WWH and the referent group are unclear, but may be due to a variety of biological factors including effects of tenofovir-based ART. The mechanisms involved in the uptake of Ca into mammary epithelial cells and its transport, packaging, and secretion into milk are complex and not completely understood (Kovacs, 2016). From mainly animal and in vitro studies—reviewed in detail elsewhere (Grinman et al., 2020; Kovacs, 2016)—the flow of ionized Ca into mammary epithelial cells is partly modulated by calcium-sensing receptors (CaSR) on the basal membranes. When extracellular Ca supply is low, reduced Ca-binding by CaSR stimulates parathyroid-related protein (PTHrP) secretion into plasma and milk. Plasma PTHrP acts to release Ca from bone and increase renal calcium conservation and thereby contributes to maintaining an adequate Ca supply to the breast. In addition, mammary CaSR and PTHrP, plus many other factors, are involved in the regulation of Ca secretion into milk. In lactating participants, milk Ca concentration is positively correlated with milk PTHrP, especially the carboxy-terminal region, suggesting a role for C-PTHrP fragments in the transport of Ca into human milk (Seki et al., 1997; Uemura et al., 1997). When extracellular Ca supply is adequate, CaSR activation downregulates PTHrP, producing a negative feedback loop. This mirrors the regulation of parathyroid hormone (PTH) by CaSR in parathyroid cells (Kumar & Thompson, 2011; Mingione et al., 2018). Alterations in CaSR modulation can lead to elevations in PTHrP/PTH. For example, in breast cancer, CaSR activation promotes, rather than reduces, PTHrP production (Grinman et al., 2020). Conversely, TDF has been shown to directly inhibit parathyroid CaSR activation and this is thought to be responsible for the elevated PTH associated with ART regimens containing TDF (Mingione et al., 2018). It is plausible, therefore, that the recent initiation of Ugandan WWH onto TDF-containing ART may have altered mammary CaSR regulation and promoted PTHrP secretion into plasma and milk, sufficient to account for the greater bone mobilization and higher milk Ca concentrations observed in the first months of lactation. More mechanistic research on calcium transport in human lactation is needed to explore this in detail, especially in the context of HIV and ART.

The strengths of the current study are that the majority of participants provided three to four milk samples across 52 weeks of lactation, and that all participants with HIV were on the same ART regimen (TDF/3TC/EFV) and reported good adherence based on pill counts. ART was provided according to the prevailing national guidelines in Uganda, which mirrored the universal ART strategy that is currently being scaled-up in Sub-Saharan Africa.

### Limitations

Milk samples were collected postpartum from 2 weeks, when most of the WWH had been on ART for about 17.6 weeks. Information on breastfeeding practices was collected based on self-reports which might be subject to recall and social desirability biases and over-estimation of exclusive breastfeeding rates. Fewer WWH than participants without HIV were primiparous, which may have introduced bias; although, mean ages were similar. Also, milk volume was not measured, and it is not possible to determine whether the differences in milk composition in the first months of lactation could have been due to a lower milk volume in WWH. However, previously, researchers have not found a relationship between milk Ca concentration and volume (Olausson et al., 2012).

## Conclusion

We have identified that milk calcium and phosphorus are altered in Ugandan WWH initiated on TDF-containing ART during pregnancy, at a time when they also experience increased bone mobilization. Further investigations are ongoing to understand the underlying mechanisms and potential implications for infant bone health and growth. Future research will be needed to consider whether these differences in calcium and phosphorus metabolism during lactation are the consequence of the human immunodeficiency virus directly, or the use of TDF-based ART. Studies will also be needed that involve women from other demographic, cultural and socioeconomic backgrounds, and those on alternative ART regimes, in order to consider the generalizability of the findings.

## Supplemental Material

sj-docx-1-jhl-10.1177_08903344221146472 – Supplemental material for Milk Calcium and Phosphorus in Ugandan Women with HIV on Tenofovir-Based Antiretroviral Therapy Supplemental material, sj-docx-1-jhl-10.1177_08903344221146472 for Milk Calcium and Phosphorus in Ugandan Women with HIV on Tenofovir-Based Antiretroviral Therapy by Florence Nabwire, Matthew M. Hamill, Mary Glenn Fowler, Adeodata Kekitiinwa and Ann Prentice in Journal of Human Lactation
