# Supplementary material for: Milk Calcium and Phosphorus in Ugandan Women with HIV on Tenofovir-Based Antiretroviral Therapy
Source: J Hum Lact. 2023 Jan 30;39(2):288–99. doi: 10.1177/08903344221146472 (PMC10115928; doi:10.1177/08903344221146472)
Supplement: sj-docx-1-jhl-10.1177_08903344221146472 – Supplemental material for Milk Calcium and Phosphorus in Ugandan Women with HIV on Tenofovir-Based Antiretroviral Therapy [file sj-docx-1-jhl-10.1177_08903344221146472.docx]

## Supplement Table 1. Summary of numbers of samples collected by group and timepoint

|  | WWH | | | | |  | | | REF | | |  | | | | Total | | | |  |  |
| --- | --- | --- | --- | --- | --- | --- | --- | --- | --- | --- | --- | --- | --- | --- | --- | --- | --- | --- | --- | --- | --- |
|  | | *n* | %≥2milks | | | |  | *n* | | %≥2milks | | |  | | *n* | | %≥2milks | | | |  |
| Participants measured in pregnancy | 100 | | |  |  | | 100 | | |  |  | | | 200 | | | |  |  | | |
| Participants who participated in lactation ^1^ | 90 | | |  |  | | 86 | | |  |  | | | 176 | | | |  |  | | |
| Participants who provided ≥2 milk samples ^2^ | 84 | | |  |  | | 81 | | |  |  | | | 165 | | | |  |  | | |
| Participants who collected milk samples at: |  | | |  |  | |  | | |  |  | | |  | | | |  |  | | |
| 1 timepoint | 4 | | |  |  | | 4 | | |  |  | | | 8 | | | |  |  | | |
| 2 timepoints | 17 | | | 20 |  | | 8 | | | 10 |  | | | 25 | | | | 15 |  | | |
| 3 timepoints | 31 | | | 37 |  | | 28 | | | 35 |  | | | 59 | | | | 36 |  | | |
| 4 timepoints | 36 | | | 43 |  | | 45 | | | 56 |  | | | 81 | | | | 49 |  | | |
| 2 weeks lactation (L2)^1^: |  | | |  |  | |  | | |  |  | | |  | | | |  |  | | |
| Attendance | 84 | | |  |  | | 78 | | |  |  | | | 162 | | | |  |  | | |
| Milk samples collected | 84 | | |  |  | | 77 | | |  |  | | | 161 | | | |  |  | | |
| Women with samples at ≥2 timepoints ^2^ | 80 | | | 95 |  | | 74 | | | 91 |  | | | 154 | | | | 93 |  | | |
| 14 weeks lactation (L14): |  | | |  |  | |  | | |  |  | | |  | | | |  |  | | |
| Attendance | 83 | | |  |  | | 81 | | |  |  | | | 164 | | | |  |  | | |
| Milk samples collected | 81 | | |  |  | | 81 | | |  |  | | | 162 | | | |  |  | | |
| Women with samples at ≥2 timepoints ^2^ | 81 | | | 96 |  | | 80 | | | 99 |  | | | 161 | | | | 98 |  | | |
| 26 weeks lactation (L26): |  | | |  |  | |  | | |  |  | | |  | | | |  |  | | |
| Attendance | 69 | | |  |  | | 72 | | |  |  | | | 141 | | | |  |  | | |
| Milk samples collected | 69 | | |  |  | | 71 | | |  |  | | | 140 | | | |  |  | | |
| Women with samples at ≥2 timepoints ^2^ | 69 | | | 82 |  | | 71 | | | 88 |  | | | 140 | | | | 85 |  | | |
| 52 weeks lactation (L52): |  | | |  |  | |  | | |  |  | | |  | | | |  |  | | |
| Attendance | 43 | | |  |  | | 56 | | |  |  | | | 99 | | | |  |  | | |
| Milk samples collected | 41 | | |  |  | | 55 | | |  |  | | | 96 | | | |  |  | | |
| Women with samples at ≥2 timepoints ^2^ | 40 | | | 49 |  | | 55 | | | 68 |  | | | 96 | | | | 58 |  | | |

*Note:* L2, L14, L26, L52 = 2, 14, 26, 52 weeks postpartum, respectively; WWH = women with HIV initiated on tenofovir-based ART during pregnancy (previously ART-naïve); REF = women without HIV; %≥2milks = percentage of participants who provided ≥2 milk samples during lactation. ^1^24 women (10 WWH and 14 REF) had exited from the study by L2 (reasons: 4 pre-term births, 5 stillbirths, 4 neonatal deaths, 5 lost to follow-up, 6 withdrawals). ^2^Data from participants who provided only one milk sample during lactation were excluded from the current analysis.

**Supplement Table 2**. Percentage differences in milk composition between the groups by timepoint for participants who gave four milk samples (rectangular data set, *N* = 81).

|  |  | | |  | |  | | | |  | |  | | | |  | |  | | | |  |
| --- | --- | --- | --- | --- | --- | --- | --- | --- | --- | --- | --- | --- | --- | --- | --- | --- | --- | --- | --- | --- | --- | --- |
|  | WWH versus REF  L2 | | |  | | WWH versus REF  L14 | | | |  | | WWH versus REF  L26 | | | |  | | WWH versus REF  L52 | | | |  |
|  | %∆ (SE) | *p* | |  | | %∆ (SE) | | *p* | |  | | %∆ (SE) | | *p* | |  | | %∆ (SE) | | *p* | |  |
|  |  |  | |  | |  | |  | |  | |  | |  | |  | |  | |  | |  |
| Ca | +9.4 (4.3) | | 0.19 | |  | | +12.4 (4.3) | | 0.04 | |  | | +5.3 (4.3) | | 0.67 | |  | | -3.6 (4.4) | | 0.88 | |
| P | +5.1 (4.2) | | 0.68 | |  | | +7.9 (4.2) | | 0.32 | |  | | +1.9 (4.2) | | 0.98 | |  | | +3.3 (4.3) | | 0.90 | |
| Ca:P ratio | +5.5 (3.7) | | 0.53 | |  | | +4.5 (3.6) | | 0.67 | |  | | +3.4 (3.6) | | 0.83 | |  | | -4.6 (3.9) | | 0.70 | |
| Na | -9.3 (10.5) | | 0.38 | |  | | +9.3 (10.6) | | 0.38 | |  | | - | | - | |  | | - | | - | |
| K | +0.8 (3.8) | | 0.82 | |  | | +3.3 (3.8) | | 0.39 | |  | | - | | - | |  | | - | | - | |
| Na:K ratio | -10.1 (10.8) | | 0.35 | |  | | +4.2 (10.9) | | 0.70 | |  | | - | | **-** | |  | | - | | **-** | |

*Note:* WWH = women with HIV initiated on tenofovir-based ART during pregnancy (previously ART-naïve) who provided a milk sample at all 4 timepoints in lactation (*n* = 36); REF= women without HIV who provided a milk sample at all 4 timepoints in lactation (*n* = 45); L2, L14, L26, L52 = 2, 14, 26, 52 weeks postpartum, respectively; Ca = calcium; P = phosphorus; Ca:P = calcium to phosphorus ratio; Na = sodium; K = potassium; Na:K = sodium to potassium ratio; milk Na and K were measured only at L2 and L14. %∆ (SE) = mean percentage difference (standard error of the mean) between the groups; the + or - signs are used to indicate the direction of between-group changes (WWH higher or lower than REF, respectively). Results were obtained from Scheffé post-hoc tests in four-timepoint hierarchical repeated-measures ANOVA models that included individual (nested by group), group, visit, and group*visit interaction.

## Supplement Table 3. Within-individual changes in milk mineral concentrations between each visit by group for participants who gave four milk samples (rectangular data set, *N* = 81).

|  |  | L2 to L14 | |  | L14 to L26 | |  | L26 to L52 | | |  |
| --- | --- | --- | --- | --- | --- | --- | --- | --- | --- | --- | --- |
|  |  | WWH  %∆ (SE) | REF  %∆ (SE) |  | WWH  %∆ (SE) | REF  %∆ (SE) |  | WWH  %∆ (SE) | REF  %∆ (SE) | | |
|  |  |  |  |  |  |  |  |  |  | | |
| Ca |  | -3.9 (4.5) | -6.9 (4.1) |  | -7.6 (4.5) | -0.5 (4.0) |  | -19.3 (4.6) ^c^ | | -10.3 (4.1) |  |
| P |  | -19.9 (4.4) ^c^ | -22.7 (4.0) ^c^ |  | -4.1 (4.4) | +1.8 (4.0) |  | -3.2 (4.6) | | -4.6 (4.0) |  |
| Ca:P ratio |  | +16.0 (3.8) ^c^ | +16.9 (3.5) ^c^ |  | -3.5 (3.8) | -2.4 (3.4) |  | -13.9 (4.0) | | -5.9 (3.5) |  |
| Na |  | -53.4 (11.0) ^c^ | -71.9 (10.0) ^c^ |  | - | - |  | - | | - |  |
| K |  | -22.3 (4.0) ^c^ | -24.8(3.6) ^c^ |  | - | - |  | - | | - |  |
| Na:K ratio |  | -31.0 (11.4) ^b^ | -45.4 (10.3) ^c^ |  | - | - |  | - | | - |  |

*Note:* L2, L14, L26, L52 = 2, 14, 26, 52 weeks postpartum, respectively; WWH = women with HIV initiated on tenofovir-based ART during pregnancy (previously ART-naïve) who provided a milk sample at all 4 timepoints in lactation (*n* = 36); REF = women without HIV who provided a milk sample at all 4 timepoints in lactation (*n* = 45); %∆ (SE) = within-individual mean percentage change between the pair of timepoints (standard error of the mean); *n(%)* = number of participants providing a milk sample at both of the respective pair of timepoints with percentage expressed relative to those who provided a sample at the earlier timepoint; Ca = calcium; P = phosphorus; Ca:P = calcium to phosphorus ratio; Na = sodium; K = potassium; Na:K = sodium to potassium ratio. Data were obtained from Scheffé post-hoc tests in four-timepoint hierarchical repeated-measures ANOVA models, that included individual identifier (nested by group), group, visit, and group*visit interaction. The + or - signs are used to indicate the direction of within-group changes (increase or decrease, respectively). All data were transformed into natural logarithms and multiplied by 100 before data analysis. Significance of four-timepoint group*visit *p*-for interaction: Ca = 0.058, P = 0.77, Ca:P = 0.22, Na = 0.21, K = 0.65, Na:K = 0.35. ^a,b,c^Significance of within-individual changes in the group obtained from the Scheffé post-hoc tests ^a^*p* ≤0.05, ^b^*p* = 0.008, ^c^*p* ≤0.001. ^A^Significance of group*visit interaction term in the two-timepoint model for the outer pair of timepoints *p* ≤0.05.
